# Infant formula containing bovine milk-derived oligosaccharides supports age-appropriate growth and improves stooling pattern

**DOI:** 10.1038/s41390-021-01541-3

**Published:** 2021-05-06

**Authors:** E. Estorninos, R. B. Lawenko, E. Palestroque, J. Lebumfacil, M. Marko, C. I. Cercamondi

**Affiliations:** 1 Asian Hospital and Medical Center, Muntinlupa City, Philippines; 2 Wyeth Nutrition, Makati City, Manila, Philippines; 3 Nestlé Product Technology Center – Nutrition, Société des Produits Nestlé S.A., Vevey, Switzerland

## Abstract

**Background:**

Adding bovine milk-derived oligosaccharides (MOS) enhances the oligosaccharide profile of infant formula. This study aimed to evaluate the safety and efficacy of a MOS-supplemented infant formula.

**Methods:**

In this double-blind randomized controlled trial, healthy infants 21–26 days old were either assigned to bovine milk-based, alpha-lactalbumin, and *sn-2* palmitate enriched infant formula (control, *n* = 115) or the same formula with 7.2 g MOS/L (test, *n* = 115) until aged 6 months. Co-primary endpoints were weight gain through 4 months and stool consistency (validated scale: 1 = watery to 5 = hard). Secondary endpoints included parent-reported GI tolerance, health-related quality of life (HRQoL), and adverse events (AEs).

**Results:**

Weight gain was similar (*p* = 0.695); the difference between test and control (mean; 95% CI: 0.29; −1.15, 1.73 g/day) was above the non-inferiority margin (−3 g/day). Test had softer stools than control (mean difference in stool consistency score: −0.31; 95% CI: −0.42, −0.21; *P* < 0.0001); fewer parental reports of harder stools (OR = 0.32, 95% CI: 0.20, 0.49; *P* < 0.0001) and less difficulties in passing stool (OR = 0.25, 95% CI: 0.09, 0.65; *P* = 0.005). Parent-reported GI tolerance and HRQoL were similar between groups as were the overall low AEs.

**Conclusions:**

MOS-supplemented infant formula is safe and well-tolerated while supporting normal infant growth and promotes softer stooling pattern without increasing parent-reported and physician-confirmed adverse health concerns.

**Impact:**

- This is the first study investigating the addition of bovine milk-derived oligosaccharides to an infant formula enriched with alpha-lactalbumin and elevated levels of *sn-2* palmitate, providing safety and efficacy data for such a formula.
- Term infant formula supplemented with 7.2 g bovine milk-derived oligosaccharides per liter supported normal infant growth, was well-tolerated and safe.
- Addition of bovine milk-derived oligosaccharides to term infant formula promoted softer stooling pattern and reduced difficulties in passing stool.
- The study shows that bovine milk-derived oligosaccharide supplemented infant formula is a safe and effective option for healthy term infants who are formula-fed.

## Introduction

Human milk is finely attuned to support infants’ optimal growth as well as overall development including gastrointestinal (GI) function and innate immunity.^1–3^ In addition to the nutritional components, human milk contains important biologically active components, such as enzymes, growth factors, antimicrobial compounds, oligosaccharides, and immunological factors.^4,5^ Emerging scientific evidence including infant studies suggests that non-digestible human milk oligosaccharides (HMOs) may provide a variety of physiologic benefits for infants such as, promoting a balanced gut microbiota,^6^ exerting antimicrobial effects,^7^ modulating immune response,^8^ reducing the incidence of infectious episodes,^9^ and also potentially enhancing cognition via the gut–brain axis.^10,11^ HMOs provide a distinctive oligosaccharide profile to human milk. Their lack in infant formula may explain the differences in health outcomes that have been associated with breastfeeding vs. formula-feeding in infants.^12^

Infant formula composition is modeled after human milk, the gold standard, and is evolving with ongoing research on human milk composition and properties as well as technological progress. A promising approach to enhance the oligosaccharide profile in infant formula moving it closer to the characteristic oligosaccharide profile in human milk is the addition of bovine milk-derived oligosaccharides (MOS). Bovine milk contains oligosaccharides that are structurally similar or identical to those found in human milk; however, in lower concentrations.^13^ Advances in technology now allow to enrich oligosaccharides from bovine milk whey and/or whey permeate at an industrial scale using a process that removes lactose and monosaccharides.^14^ MOS are primarily composed of galacto-oligosaccharides containing also inherent sialylated oligosaccharides which are structurally identical to sialylated oligosaccharides found in human milk.^15–17^ Thus, the addition of MOS originating from whey sources to infant formula moves the oligosaccharide profile closer to that of human milk and may result in certain functional benefits in formula-fed infants that are observed in breastfed infants.^16^

Previous randomized controlled trials have evaluated MOS-supplemented infant formula, with or without probiotics, and demonstrated positive physiological effects (e.g., softer stools or a bifidogenic effect).^18–22^ However, to our knowledge, no trial has evaluated the safety and efficacy of the addition of 7.2 g MOS/L to an infant formula enriched with alpha-lactalbumin and *sn-2* palmitate. To clinically demonstrate GI tolerance and potential incremental benefits of MOS in such a formula matrix is of particular importance, as higher levels of both, alpha-lactalbumin and *sn-2* palmitate, have been shown to have positive effects on the stooling pattern, digestive function, and gut microbiota during early infancy.^23–26^ The aim of this study was therefore to evaluate the safety and efficacy of an infant formula enriched with alpha-lactalbumin and *sn-2* palmitate, and further supplemented with 7.2 g MOS/L compared with an identical formula without MOS. Co-primary endpoints were infant growth through 4 months of age and stool consistency. Secondary endpoints included parent-reported GI tolerance of the formula, health-related quality of life, and parent-reported and physician-confirmed adverse events (AEs).

## Methods

### Study design

This was a double-blind, parallel-group, randomized controlled trial of healthy, term formula-fed infants recruited at the Asian Hospital and Medical Center, Muntinlupa City, Philippines between January 2016 and February 2017. Infants meeting the eligibility criteria were randomized to receive either control or test formula from study entry through 6 months of age. Randomization was carried out using the dynamic allocation algorithm in Medidata Balance (New York, NY) and was stratified by infant sex, and mode of delivery (vaginal or cesarean). Investigators, study staff, and parents/caregivers (hereafter, “parents”) were blinded to the study formulas. Formulas were coded by the manufacturer (Wyeth Nutritionals Ireland Ltd., Askeaton Co. Limerick, Ireland) using three non-speaking codes within each formula group.

The control formula was an intact-protein, bovine milk-based, whey-predominant, alpha-lactalbumin-enriched term infant formula with 13.4 g protein per liter of reconstituted formula and 45% high *sn*-2 palmitate fat blend (Betapol; Loders Croklaan, Wormerveer, the Netherlands) with the remaining fat component composed of soy oil, sunflower oil, and coconut oil. The test formula was identical to the control formula except for the addition of MOS originating from bovine milk whey permeate at a total oligosaccharide concentration of 7.2 g/L reconstituted formula. Parents were advised to feed the study formulas to their infants as they deemed appropriate, based on the infant’s appetite, age, and weight. Complementary foods were introduced when the infants reached 4 months of age.

The study was approved by the Institutional Review Board of the Asian Hospital and Medical Center, Muntinlupa City, Philippines (2015-02-A), and the Food and Drug Administration of the Philippines. Written informed consent was obtained from the parents or legal guardian of each infant before enrollment. The study was registered at clinicaltrials.gov (NCT02670863) and performed in accordance with the International Conference on Harmonization guidelines for Good Clinical Practice and the provisions of the Declaration of Helsinki and its amendments.

### Participants

Enrolled infants were healthy, term (born between 37–42 weeks gestation), singleton, aged 21–26 days (0.75 months) at enrollment, with weight-for-length and head-circumference-for-age *z*-scores within ±3 SD according to the World Health Organization (WHO) Child Growth Standards.^27^ Infants were required to have been exclusively fed and tolerated intact protein bovine milk formula at enrollment. The study endorsed breastfeeding as the optimal nutrition for infants; only infants whose parents had made the decision to formula feed prior to study screening were enrolled. Exclusion criteria included feeding with complementary foods or liquids prior enrollment, parents not expected to comply with the protocol, a medical condition or history that could interfere with the interpretation of study results, or medications/supplements known or suspected to affect fat or calcium digestion, absorption, and/or metabolism, stool characteristics, growth, or gastric acid secretion.

### Trial visits

Clinic visits were conducted at the trial enrollment (baseline visit) at 0.75 months of age, and then at 1.5, 2.5, 4, and 6 months of age. Phone calls were conducted midway between the clinic visits and at 14 days after the 6-month visit. At the baseline visit, demographics, household characteristics, and medical history were collected, a clinical examination was performed, and anthropometric measures (body weight, length, head circumference) were collected. Parents also completed a 1-day GI Symptom Record to retrospectively document stool characteristics, GI symptoms and associated behaviors, and formula intake for the day before the baseline visit. At each post-baseline visit, a clinical examination was conducted and anthropometry was obtained. Health-related quality of life (HRQoL) data was collected during the clinic visits at 2.5, 4, and 6 months of age. In addition, parents completed a 3-day GI Symptom Record at home to prospectively document stool consistency and GI tolerance indicators as well as feeding information for the 3 consecutive days prior to the post-baseline visits at 2.5, 4, and 6 months of age. Parent-reported and physician-confirmed AEs were recorded during visits and phone calls throughout the study.

### Outcome measures

The co-primary outcomes were growth velocity and stool consistency. Growth velocity was measured as mean daily weight gain in grams per day between baseline visit and the visit at 4 months of age (calculated as the difference in infant weight between the two visits, divided by the number of days between these two visits), as recommended in guidelines from the American Academy of Pediatrics Task Force on Clinical Testing of Infant Formulas.^28^ Stool consistency was reported by parents for each stool passed by the infant on the 3-day GI Symptom Record for the 3 consecutive days prior to the post-baseline visits at 2.5, 4, and 6 months of age (1-day GI Symptom Record for the baseline visit) using standardized photographs of stools corresponding to a validated 5-point stool consistency scale (1 = watery, 2 = runny, 3 = mushy soft, 4 = formed, 5 = hard). A mean consistency score was calculated for the post-baseline study period (considering all post-baseline bowel movements) as well as for the 3-day period at the post-baseline visits and for the 1-day period at baseline.

Secondary outcomes included other anthropometry measurements (weight, length, head circumference, body mass index [BMI], and corresponding *z* scores), GI tolerance indicators, formula intake, HRQoL, and occurrence of parent-reported and physician-confirmed AEs. Anthropometric measures were performed according to standard procedures. Infant weight was measured without clothing or diaper on a calibrated electronic scale (Seca 334, Hamburg, Germany) to the nearest 10 gram. Recumbent length was measured on a pediatric length board (Ellar Instrument, Washington DC) to the nearest 1 mm. Head circumference was measured to the nearest 1 mm using a non-elastic plastic-coated measuring tape (Seca 212, Hamburg, Germany). *Z*-scores for anthropometric measures were calculated according to the 2006 WHO Growth Standards.^27^ GI tolerance was assessed using data recorded by parents in the 3-day GI Symptom Record (1-day Record for baseline assessment) for stool frequency, difficulty passing stool, frequency of spitting-up/vomiting, flatulence (frequency and episodes when the flatulence made the baby fussy or uncomfortable), and episodes and duration per episode of crying/fussing and sleep. In addition, parents were asked to record the volume of formula intake, and other foods or liquids consumed other than the formula. Infant HRQoL was assessed using the standardized validated Infant and Toddler Quality of Life Questionnaire™ (ITQOL).^29^ This self-administered questionnaire was linguistically translated from English into Tagalog according to rigorous international guidelines.^30,31^ It includes 68 items that apply to infants <1 year of age assessing 9 relevant infant- and parent-focused concepts (overall health, physical abilities, growth and development, discomfort/pain, temperament and moods, general health perceptions, parent impact—emotional, parent impact—time, family cohesion). Summary scores for the infant- and parent-focused concepts were calculated with higher scores indicating better HRQoL (possible range: 0–100) and compared between the groups. In order to adjust for potential effects of the parent’s own physical and mental functioning on their perception of their infant’s quality of life, HRQoL of the parents was assessed using the Short Form 36-item Health Survey, version 2 (SF-36v2),^32^ from which 2 composite scores, the physical and mental component summary scores, are derived. Higher scores indicate better functional physical and mental health. AEs were recorded by the study staff during clinic visits and phone calls with parents throughout the study, and 14 days after completion of the feeding. All parent-reported and physician-confirmed AEs were categorized using the Medical Dictionary for Regulatory Activities (MedDRA) preferred terms.

### Statistical methods

The sample size was calculated using R 3.0.1 (2013, R Foundation for Statistical Computing, Vienna, Austria) based on growth velocity and stool consistency using a hierarchical approach prioritizing growth velocity. A non-inferiority margin of −3 grams (g)/day was used to demonstrate non-inferiority in growth velocity according to guidelines from the American Academy of Pediatrics.^28^ Assuming a standard deviation of 5.5,^26,33^ 103 infants per group would provide a power of 90% at *α* = 0.05 to show that the lower limit of the 95% two-sided confidence interval (CI) of the intervention effect (test minus control) is larger than −3 g/day. Previously published stool consistency data^19^ indicated a sample size of 103 infants per group would provide a power >90% to detect a difference between groups of 0.3 in mean stool consistency score assuming a standard deviation of 0.55. To allow for a dropout rate of 20%, 130 infants per group were planned to be enrolled. Since the actual dropout rate during the study was lower than 10%, enrollment was stopped after a total of 230 enrolled infants (115 per group).

Growth velocity (weight gain in g/day from enrollment to 4 months) was analyzed by a general linear model with the intervention group, baseline assessment, sex, and mode of delivery as covariates. Weight gain in the test group (hereafter “TG”) was considered non-inferior to the control group (hereafter “CG”) if the lower bound of the 2-sided 95% CI for the difference between groups (TG minus CG) was above the non-inferiority margin of −3 g/day. Overall post-baseline stool consistency score was analyzed by a general linear model with the same covariates as for growth velocity. As supportive analysis, stool consistency score was also analyzed using a mixed model for repeated measures (MMRM) including also visit and intervention group and visit interaction as covariates. Categorical analysis of stool consistency for all post-baseline visits together was done using an independent *t*-test and a multinomial repeated-measures logistic regression (RMLR) model with the intervention group, baseline stool consistency, sex, mode of delivery, and antibiotic use as covariates. The outcome of the logistic regression is presented as odds ratios (OR) with 95% CI indicating the odds of having stool consistency about one point higher/harder on the scale.

Stool frequency per day, duration of crying/fussing, and sleeping episodes for all post-baseline visits together were analyzed using the same model and covariates as for growth velocity. Same covariates were also used in the negative regression models for repeated measures to analyze the post-baseline incidence rate ratio (IRR) of frequency of spitting-up/vomiting and flatulence, fussiness/discomfort due to flatulence, and crying/fussing and sleeping episodes. Overall difficulty in passing stool and categorical variables of the HRQoL scales (ITQOL and SF-36v2) were analyzed using binomial and multinomial (RMLR) models with the intervention group, baseline value, visit, sex, and mode of delivery as covariates. The continuous variables of the HRQoL scales (ITQOL and SF-36v2) and anthropometric *Z*-scores were analyzed with MMRM including the same covariates as the regressions for the categorical variables plus intervention group and visit interaction. Comparisons of the percentage of infants for AE of interest, for which the number of infants with at least one reported AE was >10 when combining the two groups, were performed using Newcombe-Wilson 95% CIs. Baseline characteristics between groups were compared using the two-sample Wilcoxon test. For all statistical tests, *P* < 0.05 was considered as significant.

The co-primary outcomes were analyzed in both the full analysis set (FAS) and per-protocol (PP) populations. The secondary outcomes were analyzed in the FAS population with the exception of AEs, which was analyzed in the safety population. The FAS population included all randomized infants with bodyweight assessment at 4 months. The PP population consisted of all infants without the following protocol deviations: hospitalization for >3 consecutive or intake of unauthorized concomitant diets for ≥3 consecutive days before 4 months of age. The safety analysis set included all randomized infants with at least one documented feeding of the randomly assigned study formula.

## Results

**Fig. 1:**
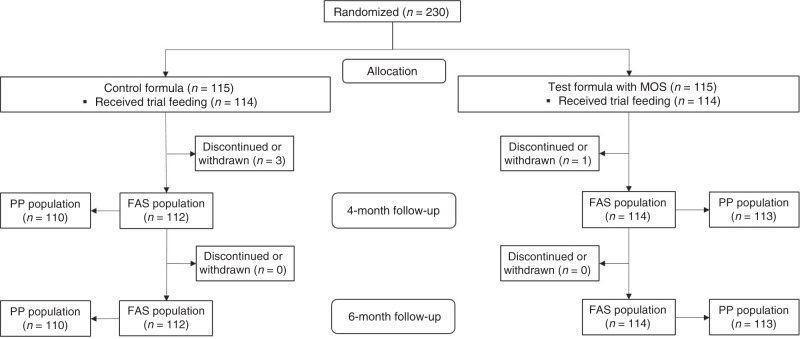
Flow of study participants. FAS full analysis set, PP per-protocol.

A total of 230 formula-fed infants were randomly assigned to receive control or test formula (115 infants per group). The flow of study participants is shown in Fig. 1. Four infants (3 in CG; 1 in TG) were withdrawn before the primary growth assessment at 4 months of age, and therefore 226 (112 in CG; 114 in TG) were included in the FAS analysis. For the PP analysis, 3 infants (2 in CG; 1 in TG) were excluded from the FAS population due to protocol deviations. Two infants (1 in each group) were excluded from the safety analysis because they discontinued without receiving any study feeding. There were no significant differences between the groups for infant age, sex, mode of delivery, and anthropometrics at enrollment. The majority of the infants in both groups (>85%) had received breast milk since birth and the average duration of breastfeeding before changing to exclusive formula-feeding was slightly longer in TG compared with CG (7.5 ± 5.3 vs. 5.9 ± 4.6, *P* = 0.028) (Table 1).

**Table 1:** Baseline characteristics of studied infants.

Infant characteristics	Control (*n* = 112)	Test (*n* = 114)
Age, days	23.3 ± 1.7^a^	23.1 ± 1.7
Sex, *n* (%) boys	58 (51.8)	59 (51.8)
Gestational age at birth, weeks	38.7 ± 0.9	38.7 ± 1.1
Mode of delivery, *n* (%) cesarean	20 (17.9)	19 (16.7)
Weight, g	3804.0 ± 406.7	3780.7 ± 433.1
Length, cm	52.1 ± 1.4	52.0 ± 1.5
Head circumference, cm	35.7 ± 1.0	35.8 ± 1.0
Ever received breast milk, *n* (%) yes	100 (89.3)	99 (86.8)
Duration of breastfeeding since birth, days*	5.9 ± 4.6	7.5 ± 5.3

*Statistically different in control vs. test group (*p* < 0.05) using two-sample Wilcoxon test.

^a^Values are mean ± standard deviation unless otherwise noted.

### Growth

**Fig. 2:**
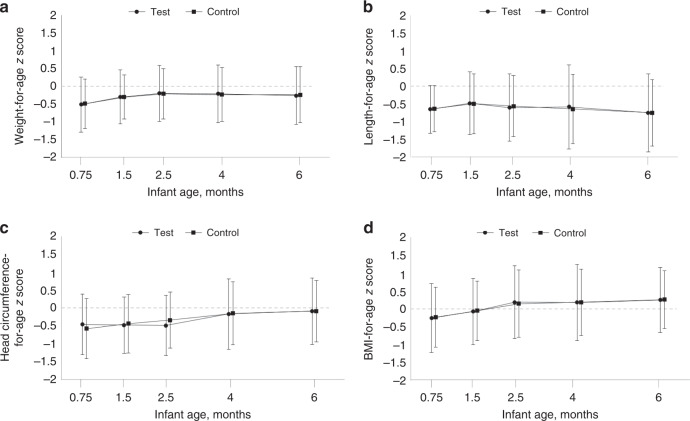
Anthropometric *z* scores from enrollment to 6 months of age based on the 2006 World Health Organization Child Growth Standards in the full analysis set (*n* = 112 in control and *n* = 114 in test). Presented *z* scores are for weight-for-age (**A**), length-for-age (**B**), head circumference-for-age (**C**), and BMI-for-age (**D**). Comparison between test vs. control group was done using a mixed model for repeated measures with assessment at baseline, visit (corresponds to the infant age in the graphs), intervention group and visit interaction, sex, and mode of delivery as covariates. No statistical differences were detected.

In the FAS population, the adjusted mean (SE) for weight gain between enrollment and 4 months of age was 29.47 (0.61) g/day for infants fed the test formula and 29.18 (0.61) g/day for those fed the control formula (Table 2). The mean difference (SE; 95% CI) in weight gain between the two groups was 0.29 (0.73; −1.15, 1.73) g/day (*P* = 0.695), with the lower limit of the 95% CI above the predefined non-inferiority margin of −3 g/day (*P* = 0.001), indicating non-inferior and comparable weight gain in infants fed the test formula compared to those fed the control formula. Results in the PP population (Table 2, mean difference 0.17 [SE: 0.73; 95% CI: −1.27, 1.61]) also demonstrated similar weight gain in the two groups. The *Z*-scores for weight-for-age, length-for-age, head circumference-for-age, and BMI-for-age from enrollment through 6 months of age are shown in Fig. 2. Overall, the mean *z*-scores in both groups tracked closely with the median of the WHO growth standards. Statistical comparisons of *z*-scores showed no significant differences between TG and CG at any visit.

**Table 2:** Comparison of weight gain from enrollment to 4 months of age between test and control group.

Population	Groups	Weight gain, g/day LS mean (SE)	Differences between groups (test − control)^a^				*P*-value for non-inferiority
			Estimate (SE)	95% CI		*P*-value	
				Lower limit	Upper limit		
FAS	Test (*n* = 114)	29.47 (0.61)	0.29 (0.73)	−1.15	1.73	0.695	0.001
	Control (*n* = 112)	29.18 (0.61)					
PP	Test (*n* = 113)	29.47 (0.60)	0.17 (0.73)	−1.27	1.61	0.815	<0.001
	Control (*n* = 110)	29.30 (0.60)					

*CI* confidence interval, *FAS* full analysis set, *LS* least squares, *PP* per-protocol, *SE* standard error.

^a^Analyzed by a general linear model with the intervention group, baseline weight, sex, and mode of delivery as covariates.

### Stool consistency

**Fig. 3:**
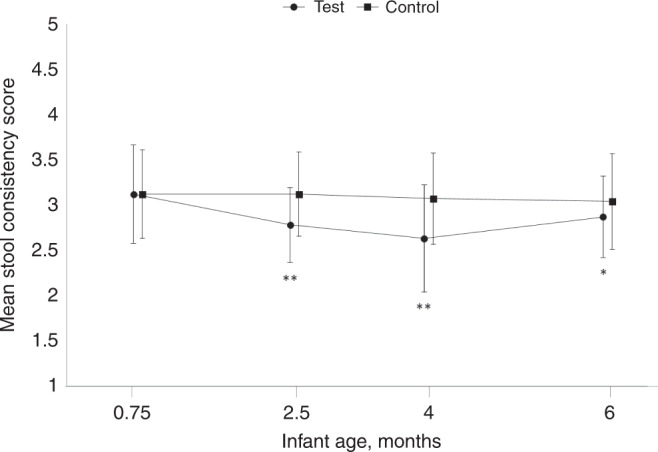
Stool consistency score (1 = watery, 2 = runny, 3 = mushy soft, 4 = formed, 5 = hard) from enrollment to 6 months of age in the full analysis set (*n* = 112 in control and *n* = 114 in test). Line graph shows mean values at different time points with the SD as whiskers. Comparison between test vs. control group was done using a mixed model for repeated measures with assessment at baseline, visit (corresponds to the infant age in the graph), intervention group and visit interaction, sex, and mode of delivery as covariates. *P < 0.05, **P < 0.001.

The mean difference (SE; 95% CI) between feeding groups (TG minus CG) in stool consistency score throughout the entire study period was −0.31 (0.05; −0.42, −0.21, *P* < 0.0001) in the FAS population and −0.32 (0.05; −0.42, −0.21, *P* < 0.0001) in the PP population, demonstrating significantly softer stools in TG than in CG. At the post-baseline visits (2.5, 4, and 6 months of age), mean stool consistency score in TG was significantly lower (indicating softer stools) than in CG as shown in Fig. 3. Further analysis of stool consistency categories showed that addition of MOS to the infant formula was associated with a decrease in the percentage of formed stool (4.1% vs. 16.8%, *P* < 0.0001) and an increase in the percentage of runny stools (21.8% vs. 9.2%, *P* < 0.0001) compared with CG. In addition, TG (vs. CG) had significant fewer parental reports of harder stools based on an average decrease of one unit in the 5-point stool consistency scale (OR = 0.32, 95% CI: 0.20, 0.49, *P* < 0.0001). Overall, the occurrence of watery stools was very low in both groups, but slightly higher in TG than in CG when considering all the post-baseline visits together (3.7% vs, 1.1%, *P* = 0.040).

### Formula intake and GI tolerance

The mean (SD) duration of consuming the study formula was 5.09 (0.06) months in TG and 5.08 (0.06) months in CG. At age 4 months, the mean (SD) volume of formula consumed was 805 (212) and 791 (212) mL/day in TG and CG, respectively. TG had significantly reduced difficulties in passing stool compared with CG (OR = 0.25, 95% CI: 0.09, 0.65, *P* = 0.005). The mean daily number of bowel movements throughout the study was slightly higher in TG than in CG (1.8 vs. 1.3, *P* < 0.0001). No significant differences were observed between the groups in the parent-reported incidences of spitting-up/vomiting, flatulence, and whether the flatulence made the baby fussy or uncomfortable (Supplemental Table 1). While the incidence of crying/fussiness reported in the study population was very low (percentage of infants with 0 crying episodes throughout all the assessment time points: 94.5% in TG; 97.5% in CG), the overall parent-reported incidence of crying/fussiness episodes throughout the study was statistically higher in TG vs. CG (IRR = 2.36, 95% CI: 1.01, 5.51, *P* = 0.048). However, the duration of crying/fussiness episodes per day was not significantly different between groups (LS mean ± SE: 8.2 ± 2.3 min in TG; 9.7±2.3 min in CG, *P* = 0.678). Similarly, the overall incidence of sleeping episodes was lower in TG compared with CG (IRR = 0.95, 95% CI: 0.91–0.99, *P* = 0.023) with no difference in the duration of sleep episodes (LS mean ± SE: 160.5 ± 3.2 min in TG; 155.9 ± 3.2 min in CG, *P* = 0.241).

### Health-related quality of life

Infant HRQoL scores were not significantly different between groups throughout the study, for either infant-focused concepts (LS mean score difference ± SE: 1.1 ± 0.74, 95% CI: −0.35, 2.56, *P* = 0.136) or parent-focused concepts (−0.42 ± 1.62, 95% CI: −3.62 to 2.78, *P* = 0.796) (Supplemental Table 2). ITQOL scores for all 9 concepts were ≥75 (available range 0–100) at any visit across both groups. Parental HRQoL measures were not significantly different between two groups over the study period, for either the physical health component (LS mean score difference ± SE: 2.18 ± 1.54, 95% CI: −0.86 to 5.22, *p* = 0.159) or the mental health component (2.39 ± 1.26, 95% CI: −0.08 to 4.87, *P* = 0.058) from SF-36v2 (Supplemental Table 2).

### Parent-reported and physician-reported adverse events

The overall number of infants who had at least one reported AE was similar in both groups (95 in TG; 93 in CG). In infants who experienced AEs, 99.5% (100% in TG) were unrelated to study feedings, and 88.9% were mild in intensity. Upper respiratory tract infection (59 and 57 infants in TG and CG, respectively), lower respiratory tract infection (24 infants in TG and CG), and post-vaccination syndrome (48 and 45 infants in TG and CG, respectively) were the most commonly reported AEs with similar incidences in both groups as confirmed by Newcombe-Wilson 95% CIs (Supplemental Table 3). GI-related AEs were similar between groups and reported in 16 infants overall: 9 in TG and 7 in CG. They included abdominal pain (1 in TG; 0 in CG), mild diarrhea (4 in TG; 1 in CG), constipation (0 in TG; 3 in CG), and vomiting (4 in TG; 3 in CG) (Supplemental Table 3). Except for one case of constipation probably related to the study product in CG, all other GI-related AEs were unrelated to the study products. There were no reports of colic. No infant in TG had a serious adverse event (SAE), while two infants in CG had a SAE of pneumonia.

## Discussion

To our knowledge, this was the first trial to evaluate the safety and efficacy of an alpha-lactalbumin and *sn-2* palmitate enriched infant formula supplemented with 7.2 g MOS per liter reconstituted formula. Previously studied formulas supplemented with the same MOS ingredient did not contain alpha-lactalbumin and elevated levels of *sn-2* palmitate, contained often probiotics, and were with different concentrations of MOS (6, 8, or 10 g/L reconstituted formula).^18–22^ Our data are, therefore, the first to demonstrate that such an infant formula supports age-appropriate infant growth, tracking closely among the median of the WHO growth standards, and promotes softer stooling pattern without parent-reported or physician-confirmed concerns of adverse health outcomes, such as watery stool or diarrhea. Additionally, secondary outcomes of parent-reported GI tolerance and parent-reported and physician-confirmed AEs were generally comparable between the groups and indicate goodtolerability of the MOS-supplemented formula.

The non-inferior growth in the TG vs. CG in accordance with American Academy of Pediatrics recommendations^28^ and the anthropometric *z*-scores showing normal growth of the infants are in agreement with the previous trials of MOS-supplemented formula with or without probiotics. In these studies, the growth parameters of healthy infants, including those born to HIV+ mothers, were not significantly different between the MOS and control groups and were aligned with WHO growth standards in the first year of life.^18–22^ Our results demonstrate that alpha-lactalbumin-enriched infant formula with elevated levels of *sn-2* palmitate and 7.2 g MOS/L provides adequate nutrition to support normal infant growth.

We found a significantly softer stooling pattern among infants fed the formula with MOS which was accompanied by a minor increase in stool frequency (1.8in TG; 1.3in CG). Parent-reported and physician-confirmed GI-related AEs demonstrated a low incidence of diarrhea, with all diarrhea episodes reported as mild and unrelated to the study product. This is important as it ensures that the softer stools observed with MOS were not associated with parental concerns regarding the incidence of diarrhea. Parent-reported stooling data demonstrating significantly fewer formed stools and fewer difficulties in passing stool in TG is further confirming the softer stooling pattern among infants fed the MOS-supplemented formula. Our data is consistent with that of the previous reports of infant formula supplemented with probiotics and MOS at levels 6–10 g/L, which showed fewer hard stools and/or improved stool consistency, approaching the stooling pattern of breastfed infants, compared with a control formula.^18–22^ Collectively, this data indicates that MOS promotes softer stools and may help prevent hard stools and constipation. These improvements are important as parameters of GI tolerance/stool pattern in formula-fed infants are often a concern for caregivers and pediatricians. A survey of 195 mothers of infants aged 3–12 weeks reported that significantly more formula-feeding mothers had concerns about stool hardness than breastfeeding mothers, resulting in both increased use of health care resources and more dietary interventions.^34^ Also, the European Food Safety Authority has recognized that changes in bowel function including softer stools are a beneficial physiological effect in infants, provided that the infants do not experience diarrhea.^35^ Our results indicate that such improvement in formula-fed infants can be achieved by adding MOS to infant formula. The effect of MOS on stooling characteristics observed in our study is an incremental effect to *sn-2* palmitate which is known to have a beneficial effect on stooling pattern, such as softening stool.^23,25,26^ MOS are prebiotic oligosaccharides whose fermentation by colonic bacteria can lead to augmented microbial mass followed by increased fecal water content, which results in softer stools. The potential selective fermentation and growth of lactobacillus and bifidobacteria species and subsequent production of short-chain fatty acids can also increase the water content of the fecal mass, and short-chain fatty acids may also stimulate gastrointestinal motility by inducing phasic and tonic contractions in circular muscles.^36^

The addition of 7.2 g MOS/L to infant formula had no effect on the parent-reported incidence of spitting-up/vomiting or flatulence in our study. We found that the parent-reported incidence of crying/fussing episodes in TG was statistically higher than that of CG. However, the overall incidence of crying reported in this study population was low; the proportion of days with no episodes of crying/fussing was above 94% for both groups, which may reflect Filipino cultural customs (i.e., believing in more constant and intense physical closeness to the young child and considering the cry-it-out method neglectful). In addition, the mean duration per crying/fussing episodes was similar between the two groups, and there was no increased parental alarm and concern about crying via AE reporting throughout the study. Therefore, the slightly increased rate of infant crying/fussiness in the MOS group was unlikely to be of clinical relevance or related to feeding intolerance or gut discomfort. It is worth noting that one of the previous MOS trials identified a higher incidence of physician-diagnosed colic among infants fed MOS-containing formula relative to the control formula.^19^ The authors suggested that the increase may have been related to the level of oligosaccharides added to the formula (10 g/L reconstituted formula), which was higher than that in other previous studies (6–8 g/L reconstituted formula)^18,20–22^ or the current study. No colic has been reported as AE in our study indicating that colic is not a concern when MOS is added to infant formula at a level of 7.2 g/L reconstituted formula.

Infant HRQoL is a broad concept that encompasses aspects of physical, psychological, and social function. Infant feeding regimen may indirectly impact infant HRQoL via GI tolerance; therefore, we evaluated it using the validated ITQOL. Mean ITQOL scores for all 9 categories of questions were ≥75 (available range 0–100) across both groups, which was consistent with similarly high scores (>75) reported in an infant study in China.^37^ One possible explanation for not seeing a difference between the groups in our study is that the impact of feeding regimen on infant overall well-being may be substantially attenuated in the presence of other factors with a stronger impact on quality of life than feeding as for example social conditions and home environment. Parental HRQoL summary scores using the SF-36v2 revealed no difference between groups, indicating little confounding influence of parental HRQoL on the perception of their infant’s HRQoL in our study.

The strengths of the present study include its novelty as the first randomized controlled trial evaluating the safety and efficacy of 7.2 g MOS/L added to an alpha-lactalbumin enriched infant formula with elevated levels of *sn-2* palmitate, as most previous trials did not investigate MOS alone but rather as part of a probiotic mix and in different formula matrices.^18–22^ Thus, this study provides a more direct evaluation of the effect of MOS with greater generalizability of the efficacy of MOS. Additionally, the trial was sufficiently powered to assess safety and efficacy co-primary outcomes and had a very low dropout rate. The randomized double-blind study design reduced the risk for systematic bias of under- or over-reporting of the parent-reported stool consistency and GI tolerance indicators, and proper instruction of the parents by study staff on how to complete the GI symptom diaries and a pictorial presentation of stool consistency mitigated the mischaracterization of these parameters. Our study also has limitations. Infants were followed through six months of age; although this was a sufficient period of time to evaluate the outcomes of interest, a longer follow-up time (i.e., up to 1 year of age) would have allowed us to evaluate potential sustained or long-term effects of MOS beyond early infancy. Our study was conducted at a single center in a single country, and some results (e.g., crying/fussing) may be influenced by country-specific cultural aspects and child-caring practices.

To conclude, this study demonstrated that infant formula supplemented with MOS at 7.2 g/L is safe and well-tolerated and supports normal age-appropriate infant growth according to WHO standards. The addition of MOS to an alpha-lactalbumin-enriched infant formula with elevated levels of *sn-2* palmitate promoted a softer stooling pattern compared with the control formula without parent-reported or physician-confirmed adverse health concerns. Such an infant formula will provide a safe and effective option for healthy term infants who are formula-fed.

## Supplementary information

Supplementary Information
