# Supplementary material for: Infant formula containing bovine milk-derived oligosaccharides supports age-appropriate growth and improves stooling pattern
Source: Pediatr Res. 2021 May 6;91(6):1485–92. doi: 10.1038/s41390-021-01541-3 (PMC9197766; doi:10.1038/s41390-021-01541-3)
Supplement: Supplementary file 1 — Supplementary Information [file 41390_2021_1541_MOESM1_ESM.docx]

**Supplemental Table 1. Group comparison of gastrointestinal tolerance variables in the full analysis set**

|  | **IRR**  **(Test vs. Control)** | **95% CI** | |
| --- | --- | --- | --- |
|  |  | **Lower limit** | **Upper limit** |
| Spitting-up/Vomiting | 1.0828 | 0.8727 | 1.3436 |
| Flatulence | 1.0155 | 0.9183 | 1.1230 |
| Fussiness/discomfort due to flatulence | 1.1766 | 0.6278 | 2.2049 |
| Crying/Fussing episodes | 2.3571* | 1.0084 | 5.5093 |
| Sleeping episodes | 0.9525* | 0.9134 | 0.9932 |

CI, confidence interval; IRR, incidence ratio rate. IRR is computed based on all post-baseline visits using negative regression models for repeated measures with intervention group, assessment at baseline, sex, and mode of delivery as covariates; *p<0.05.

**Supplemental Table 2. Group comparison of infant- and parental-related health related quality of life (HRQoL) measures in the full analysis set**

|  | **LS mean difference/OR^a^**  **(Test – Control)** | **Standard error** | **95% CI** | |
| --- | --- | --- | --- | --- |
|  |  |  | **Lower limit** | **Upper limit** |
| **Infant-related HRQoL, ITQOL** |  |  |  |  |
| **Infant-focused concepts** | **1.1** | **0.74** | **-0.35** | **2.56** |
| Overall health | OR: 1.07 | 0.21 | 0.73 | 1.57 |
| Physical abilities | -1.02 | 2.51 | -6.20 | 4.17 |
| Growth and development | 0.33 | 0.89 | -1.42 | 2.08 |
| Bodily pain/discomfort | -0.68 | 1.20 | -3.05 | 1.69 |
| Temperament and moods | 0.89 | 1.09 | -1.25 | 3.03 |
| General health perceptions | 1.7 | 1.30 | -0.86 | 4.25 |
| **Parent-focused concepts** | **-0.42** | **1.62** | **-3.62** | **2.78** |
| Parent impact – emotional | -0.94 | 2.64 | -6.14 | 4.25 |
| Parent impact – time | 0.57 | 2.01 | -3.39 | 4.53 |
| Family cohesion | OR: 1.15 | 0.23 | 0.78 | 1.69 |
| **Parent-related HRQoL, SF-36v2** |  |  |  |  |
| **Physical health** | **2.18** | **1.54** | **-0.86** | **5.22** |
| Physical health, physical functioning | 0.60 | 2.09 | -3.52 | 4.72 |
| Physical health, role limitations due to physical health | 1.97 | 2.25 | -2.46 | 6.40 |
| Physical health, bodily pain | 2.71 | 2.24 | -1.71 | 7.12 |
| Physical health, general health | 1.56 | 1.51 | -1.42 | 4.54 |
| **Mental health** | **2.39** | **1.26** | **-0.08** | **4.87** |
| Mental health, vitality | 0.43 | 1.52 | -2.58 | 3.43 |
| Mental health, social functioning | 2.23 | 1.82 | -1.36 | 5.82 |
| Mental health, role-emotional | 2.56 | 2.29 | -1.95 | 7.07 |
| Mental health, mental health | 0.74 | 1.30 | -1.82 | 3.29 |
| Mental health, reported health transition | OR: 0.71 | OR: 0.14 | 0.48 | 1.04 |

CI, confidence interval; ITQOL, Infant and Toddler Quality of Life Questionnaire; OR, odds ratio; SF-36v2, Short Form 36-item Health Survey, version 2.

^a^ Categorical variables were analyzed using multinomial logistic regressions for repeated measures with intervention group, baseline value, visit, sex, and mode of delivery as covariates. Continuous variables were analyzed with a mixed model for repeated measures including the same covariates as the regressions plus visit and intervention arm and visit interaction. No variables were different between groups (all p>0.05).

**Supplemental Table 3. Incidence of overall and major adverse events as well as gastrointestinal-related adverse events in the safety set**

| **Adverse event (AE)** | **Control (n = 114)** | **Test (n = 114)** | **Newcombe-Wilson**  **95% CIs^a^** |
| --- | --- | --- | --- |
| Any AE | 93 (81.6)^b^ | 95 (83.3) | 1.8% (-8.2% to 11.7%) |
| Upper respiratory infection^c^ | 57 (50.0) | 59 (51.8) | 1.8% (-11.0% to 14.5%) |
| Lower respiratory tract infection^d^ | 24 (21.1) | 24 (21.1) | 0.0% (-10.6% to 10.6%) |
| Post vaccination syndrome | 45 (39.5) | 48 (42.1) | 2.6% (-10.0% to 15.1%) |
| Gastrointestinal-related AEs | 7 (6.1) | 9 (7.9) | 1.8% (-5.3% to 8.9%) |
| Abdominal pain | 0 (0) | 1 (0.9) | - |
| Diarrhea | 1 (0.9) | 4 (3.5) | - |
| Constipation | 3 (2.6) | 0 (0) | - |
| Vomiting | 3 (2.6) | 4 (3.5) | - |

^a^ Newcombe-Wilson 95% CIs were used to compare the percentage of subjects for specific AEs of interest that were reported in >10 infants. None of the AEs were different between groups.

^b^ n (%), number (percentage) of infants who had at least one reported AE, all such values.

^c^ Upper respiratory infection (comprised of the following PTs: respiratory tract infection, acute tonsillitis, laryngitis, pharyngitis, rhinitis, tracheitis, upper respiratory tract infection, respiratory tract infection viral, viral upper respiratory tract infection, nasal obstruction, and nasopharyngitis).

^d^ Lower respiratory tract infection (comprised of the following PTs: bronchitis, lower respiratory tract infection, pneumonia, respiratory syncytial virus bronchiolitis, respiratory syncytial virus bronchitis, bronchiolitis, lower respiratory tract infection viral, respiratory tract infection viral).
